# Dual effect of oxidative stress on leukemia cancer induction and treatment

**DOI:** 10.1186/s13046-014-0106-5

**Published:** 2014-12-18

**Authors:** Udensi K Udensi, Paul B Tchounwou

**Affiliations:** NIH/NIMHD RCMI Center for Environmental Health, College of Science, Engineering and Technology, Jackson State University, Jackson, 39217 MS USA

**Keywords:** Oxidative stress, Leukemia, Antioxidants, Reactive oxygen species, Treatment, Apoptosis

## Abstract

Oxidative stress (OS) has been characterized by an imbalance between the production of reactive oxygen species (ROS) and a biological system’s ability to repair oxidative damage or to neutralize the reactive intermediates including peroxides and free radicals. High ROS production has been associated with significant decrease in antioxidant defense mechanisms leading to protein, lipid and DNA damage and subsequent disruption of cellular functions. In humans, OS has been reported to play a role in the pathogenesis of neurodegenerative diseases such as Alzheimer’s disease, Huntington’s disease, Lou Gehrig’s disease, multiple sclerosis and Parkinson’s disease, as well as atherosclerosis, autism, cancer, heart failure, and myocardial infarction. Although OS has been linked to the etiology and development of chronic diseases, many chemotherapeutic drugs have been shown to exert their biologic activity through induction of OS in affected cells. This review highlights the controversial role of OS in the development and progression of leukemia cancer and the therapeutic application of increased OS and antioxidant approaches to the treatment of leukemia patients.

**Electronic supplementary material:**

The online version of this article (doi:10.1186/s13046-014-0106-5) contains supplementary material, which is available to authorized users.

## Introduction

Despite the numerous scientific studies on leukemia, there are still gaps on information about the risk factors and actual causes and such knowledge are necessary for effective treatment and prevention strategies to be established. OS is a cellular environmental condition that results from excessive production of ROS with reduced or lack of antioxidants production to maintain homeostasis [1]. Although OS is known to induce cancer, it has also been reported to have beneficial attributes. For instance it induces apoptosis which is a mechanism in cancer treatment [2]. ROS are essential signaling molecules which play different important roles in cellular processes such as promoting health and longevity [3] and antimicrobial phagocytosis by cells of the innate immune system [4]. Over production of reactive oxygen species without adequate response by the innate antioxidant system to maintain the balance leads to an OS environment. Some of the ways OS and free radicals are created are through chemotherapeutic agents and radiation therapy which generate reactive ROS in patients during cancer therapy. The interaction between growing cancer cells and the host immune response also generate OS. The effect of OS to the cells may either be acute or chronic. Chronic OS results from little oxidative damage which accumulates during the life cycle of the cell and subsequently disrupts essential cellular functions and triggers many cancers [5]-[7], including solid tumors such as prostate carcinoma [8], melanoma [9], and several hematopoietic malignancies such as acute lymphoblastic leukemia (ALL), [10] myelodysplastic syndrome (MDS), [11] and myeloid leukemia; chronic myeloid leukemia (CML) and acute myeloid leukemia (AML) [12]. OS has also been implicated in other human diseases including acute respiratory distress syndrome [13], aging [14], Alzheimer [15], atherosclerosis [16], cardiovascular diseases, and amyotrophic lateral sclerosis [17], diabetes [18], inflammation [19], inflammatory joint disease [20], neurological disease [21], obesity [22], Parkinson [23], pulmonary fibrosis [24], Rheumatoid arthritis [25], and vascular disease [26].

In some cancers ROS facilitate carcinogenesis by protecting the cell from apoptosis and promoting cell survival [27],[28], inducing cell proliferation [29], migration and metastasis [30] and drug-resistance [31]. The regulation of OS is an important factor in both tumor development and responses to anticancer therapies. Many signalling pathways that are linked to tumorigenesis can also regulate the metabolism of reactive oxygen species (ROS) through direct or indirect mechanisms [32]. Depending on the condition and environment, OS may influence the role of ROS to either benefit malignant cancer formation or cancer treatment. This review explores the role of OS in leukemogenesis and highlights its importance in leukemia treatment. Understanding the molecular mechanisms of oxidative will help in developing new and reliable treatment and preventive measures for the different types of leukemia.

### Oxidative stress

**Figure 1:**
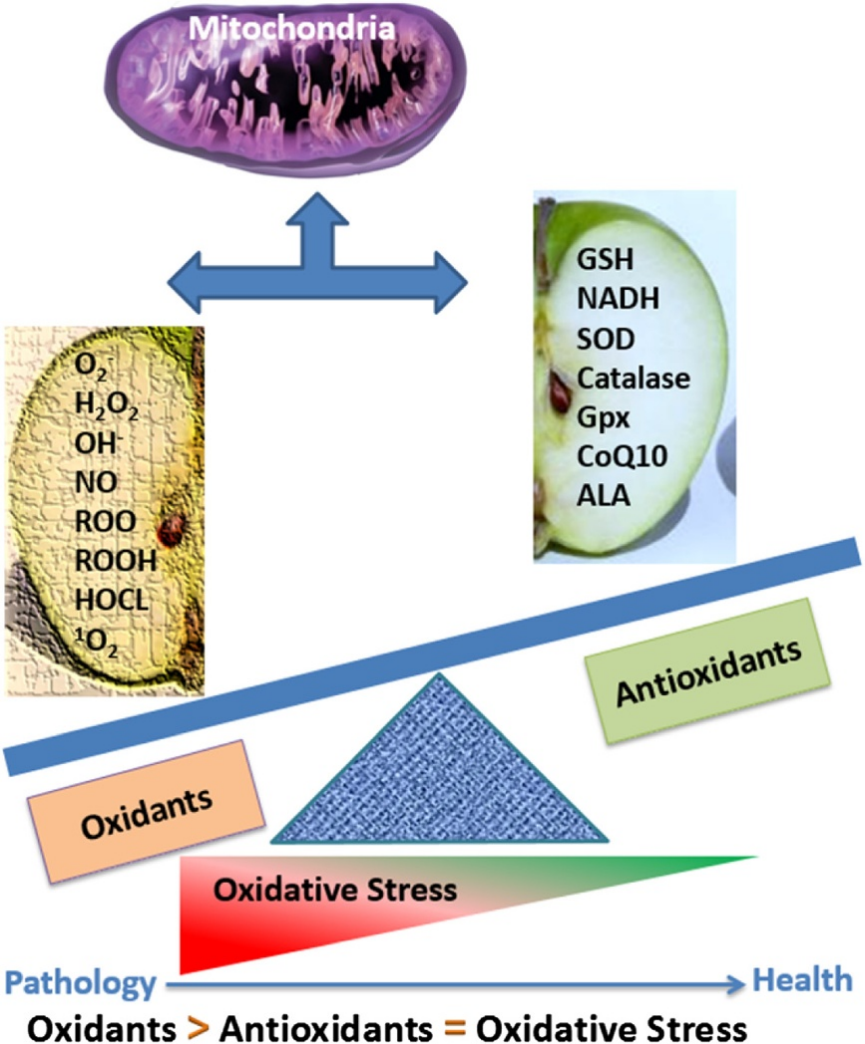
**Types of oxidants and antioxidants which imbalance will lead to oxidative stress (OS).** OS is described as an excess production of ROS compared to antioxidant defense.

OS results when there is an imbalance between the generation of oxygen-free radicals or reactive oxygen species (ROS) and response from the antioxidant defense systems [33]. Free radicals are molecules or molecular fragments that contain one or more unpaired electrons which make them highly reactive [34]. Under normal circumstances the effect of reactive species is balanced by the antioxidant action of enzymatic and non-enzymatic antioxidants. Essential cellular functions, such as gene expression, are influenced by the balance between pro- and antioxidant conditions [35]. Antioxidant defenses are extremely important as they represent the direct removal of free radicals (pro-oxidants), providing maximal protection for biological sites. Physiological processes of the cell including cellular proliferation and host defense may be interrupted when the ROS exceed or antioxidants fall below the homeostatic set point as illustrated in Figure 1. Increased ROS could be detrimental and cause cell death or accelerate ageing and age-related diseases. ROS contribute to cellular dysfunction and cell death by damaging proteins, lipids, and DNA. They may also serve as stress signals which activate specific redosensitive signaling pathways [36]. Production of ROS, reactive oxygen intermediate (ROI) and reactive nitrogen intermediate (RNI) are part of human body’s physiological processes [37]. Both ROI and RNI activities affect cells in similar ways, thus, the concept of OS has been expanded to include; hydroxyl and superoxide radicals, hydrogen peroxide and singlet oxygen, reactive nitrogen species (RNS) or RNI including nitric oxide (NO), peroxynitrite and, S-nitrosothiols [38].

Naturally, the human body maintains the redox balance by the combined action of antioxidant enzymes which include superoxide dismutase (SOD), catalase (CAT), glutathione (GSH), glutathione peroxidase (GPx) and glutathione reductase, monoamine oxidase (MAO), ascorbate, α-tocopherol, cysteine, thioredoxin, and vitamins [33],[39]. Coenzyme Q10 (CoQ10) is a very important endogenous antioxidant [40]. Also crucial are the peroxiredoxins (Prx), a family of small non-seleno peroxidases that regulate cellular ROS [41]. In their study, Valko and co-workers observed that ROS generated by the mitochondria, nicotinamide adenine dinucleotide phosphate (NADPH) oxidases impacted on cell-cycle progression, cell motility, and growth factor signaling in a variety of normal cell types [4]. More ROS are produced when there is irregularity with the body’s antioxidant defense system due to disease other negative environmental conditions [39]. For example, ROS production spikes as cells transition from normal tissue to invasive carcinoma caused by the increase in metabolic aberrations in the transforming cells [42]. Also, levels of oxidants increase with corresponding decrease in levels of antioxidants in leukemia [43]. This view is supported by a study on ALL patients which showed that a tilt in ROS/antioxidant balance led to increase in serum level of markers of OS such as thiobarbituric acid reactive substances (TBARS), and serum protein carbonylation in ALL patients than in normal controls [10]. Literature is replete with reports on the harmful effects of ROS, however, some of the harmful concept are being exploited as potential targets for drug development [34]. For example low concentrations of ROS was observed to induce mitogenic response and trigger a series of cellular responses during noxia which helps to fight against infectious. But higher concentrations of ROS may induce damage of cell structures, including lipids and membranes, proteins and nucleic acids [44].

### Sources/Activators of Reactive Oxygen Species (ROS)

**Figure 2:**
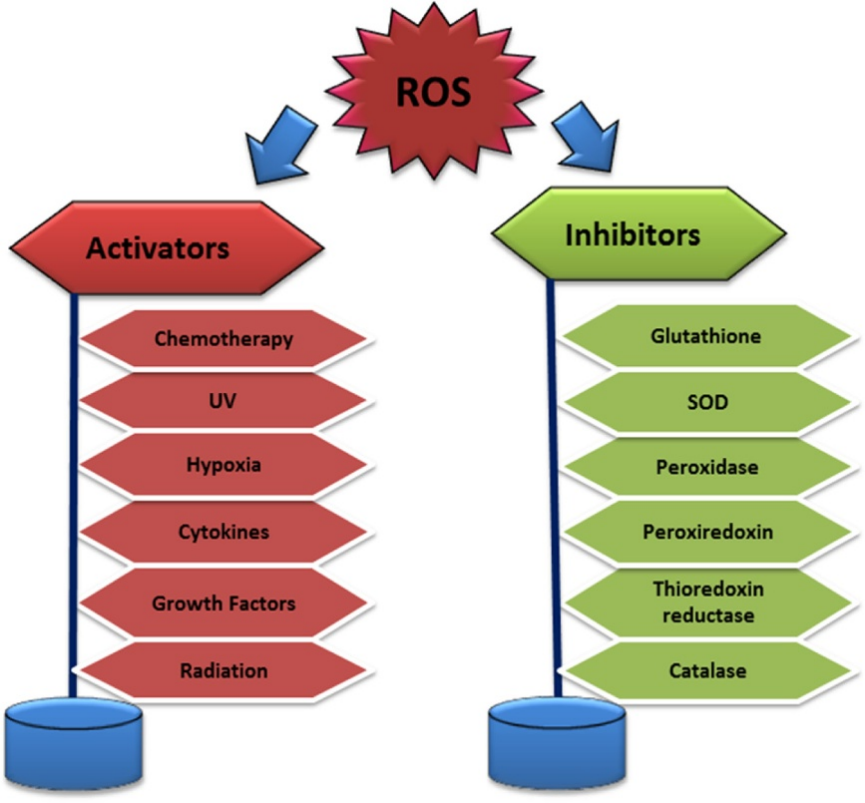
**Schematic representation of various activators and inhibitors of reactive oxygen species production**[53]**.**

ROS production can be triggered by both endogenous and exogenous factors. The endogenous sources are from cellular metabolism especially during mitochondria-catalyzed electron transport reactions, cytochrome P450 metabolism, and activities of peroxisomes, neutrophils, eosinophils and macrophages during inflammation [45]. Activated macrophages can initiate an increase in oxygen uptake which triggers increases in different reactive oxygen species, such as superoxide anion, nitric oxide and hydrogen peroxide [46]. Animal Cytochrome P_450_ enzymes is suspected to have a dual role of providing protection against natural toxic chemicals from plants and conversely inducing the production of reactive oxygen species especially superoxide anion and hydrogen peroxide after the breakdown or uncoupling of the P450 catalytic cycle [47]. Exogenous sources may include exposure to various xenobiotics, irradiation by UV light, X-rays and gamma-rays. And metal-catalyzed reactions that produce free radicals, chlorinated compounds, and barbiturates [48]. Mitochondria are regarded as the most important physiological source of radicals in living organisms because it generates approximately 2–3 nmol of superoxide/min per mg of protein [45]. Superoxide radical is considered as the stoichiometric precursor for hydrogen peroxide (H_2_O_2_) [49]. Ubisemiquinone is a reductant of oxygen in mitochondrial membranes [45]. Other endogenous sources of superoxide radical besides mitochondria include xanthine oxidase (XO), an enzyme that is found within the various tissues of mammals and which can be acquired from bacteria [50]. XO catalyzes the conversion of hypoxanthine to xanthine and xanthine to uric acid reducing molecular oxygen to form superoxide anion which subsequently produces hydrogen peroxide [34]. During their normal catalytic process, enzymes found in the peroxisomes can produce hydrogen peroxide (H_2_O_2_), superoxide (O_2•_^−^), or nitric oxide (NO_•_) which can readily react to form other ROS and RNS such as peroxynitrite (ONOO^−^), hydroxyl radical (_•_OH), and alkyl peroxides (ROOH). Peroxisomes play vital roles in fat metabolism especially in the liver and other organs leading to the production of H_2_O_2_, but not O_2•_^−^ mostly after prolonged starvation [51],[52]. Superoxide may act as a reductant or an oxidant and is a key molecule in several subsequent physiologic reactions. Most of the superoxide generated in vivo is converted into H_2_O_2_ primarily by the actions of superoxide dismutases, which exist in cytosolic (SOD1), mitochondrial (SOD2), and extracellular (SOD3) isoform [51]. Activators and inhibitors of ROS are illustrated in Figure 2.

### Mechanisms of action of ROS

**Figure 3:**
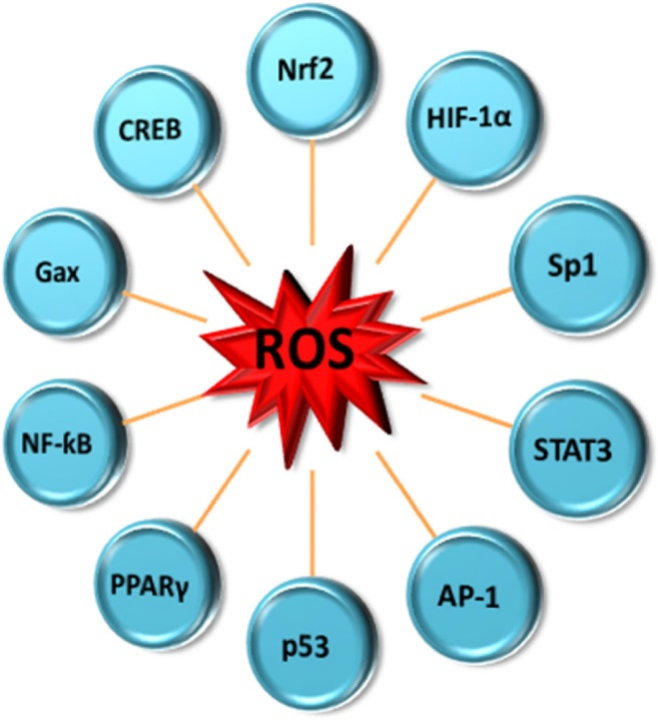
**Transcription factors that are modulated by reactive oxygen species**[53],[54]**.**

There are different mechanisms through which ROS cause cell damage. For instance endogenous damage occurs when intermediates of oxygen (dioxygen) reduction - oxygen-free radicals (OFR) attack the bases and the deoxyribosyl backbone of DNA. This endogenous DNA damage demonstrates the genotoxic, carcinogenic, and mutations induction properties of ROS [34]. Another important mechanism is the perturbation of transcription factors activities. Understanding the complex redox regulation of the transcription of specific eukaryotic genes will give more insight into the role of redox-sensitive transcription factors in this process. Figure 3 shows a schematic diagram of transcription factors that are modulated by ROS. The hypoxia-inducible transcription factor HIF-1 mediates upregulation of plasminogen activator inhibitor-1 (PAI-1) expression under low oxygen tension (hypoxia) and promotes angiogenesis [54]. Self-renewal of hematopoietic stem cells (HSCs), leukemia-initiating cells (LICs) and myeloproliferative neoplasms occurs under hypoxic condition [55]. There are contrary opinions on the role of HIF-1 in leukemia pathogenesis but it induces genes that facilitate adaptation and survival of cells and the whole organism when the environment changes from normoxia (∼21% O_2_) to hypoxia (∼1% O_2_). Cells and tissues may also be protected from oxidative damage by NF-E2 related factor 2 (Nrf2), a member of the cap‘n’collar family of basic leucine zipper transcription factors which induces activation of genes encoding detoxifying and antioxidant enzymes [56]. Activator protein 1 (AP-1) regulates diverse biological functions, including cell proliferation, protein synthesis, apoptosis and secretion of inflammatory and profibrotic factors. AP-1 also has a double edge function in tumorigenesis in that it can have both oncogenic or tumor suppressive activity depending on the cell context and the genetic background of the tumor [57],[58]. Other important genes involved in the linkage between OS and leukemia are matrix metallopeptidase 1 (MMP-1) and endothelin-1 which are expressed in response to Angiotensin II (Ang II) [59]. The growth arrest homeobox gene, mesenchyme homeobox 2 (Gax or MEOX2) regulates cell differentiation, proliferation, and migration and Gax is likely to have a regulatory function in the G0-to-G1 transition of the cell cycle in vascular smooth muscle cells [60],[61]. Cyclic AMP response element-binding protein (CREB) may have pro-survival effects on APL cells by protecting blasts from APL patients with acute promyelocytic leukemia (APL) against death induced by first-line anti-leukemic anthracyclines like daunorubicin (DNR) [62].

Peroxisome proliferator-activated receptor gamma (PPARγ) is involved in pathological processes of different disease and it is a potential therapeutic target for the treatment of a diverse array of disorders including AML, in which supplementary treatment is suggested to include deregulation of the PPARγ signaling [63]. The ability of the tumor suppressor p53 under stress to differentially regulate its target genes which regulate cell-cycle arrest, apoptosis or senescence of hematopoietic stem cell (HSC) could be a possible treatment target for leukemia [64]. However, there is progress in the development of drugs based on OS mechanism. Some are on clinical trial stage, for instance a potent NF-kB inhibitor, Deferasirox is on clinical trial for the treatment of AML and MDS [65]. Another target of interest is the antiapoptotic transcription factor, signal transducer and activator of transcription 3 (STAT3) which is modulated by OS. STAT3 is activated when spleen tyrosine kinase (SYK) is induced in an ecdysone-inducible mammalian expression system. The link between SYK and STAT3 is being explored as a potential target for therapeutic purposes in human B-lineage leukemia/lymphoma cells [66].

### Sources/activators of antioxidants

To counter the deleterious effects of free radicals and OS in the organelle, mitochondria also harbor antioxidants including GSH and enzymes, (superoxide dismutase (SOD) and glutathione peroxidase (GPx),) which are present on both sides of their membranes [67]. Antioxidants could be either endogenous or exogenous. The endogenous antioxidants produced mostly in the mitochondria include superoxide dismutase, (SOD), alpha lipoic acid (ALA), Coenzyme Q10 (CoQ10), catalase (CAT), and glutathione peroxidase (Gpx), glutathion (GSH), ferritin, uric acid, bilirubin, metallothioneine, L-carnitine and melatonin [68]. Exogenous antioxidants are acquired from diet such as vitamin E (α-tocopherol) which is present in vegetable oil and wheat germ. Vitamin E can prevent lipid peroxidation of plasma membrane because it is lipid soluble [69]. Other important exogenous antioxidants found in plants (fruits, vegetables, medicinal herbs) include phenolic compounds (phenolic acids, flavonoids, quinones, coumarins, lignans, stilbenes, tannins etc.), nitrogen compounds (alkaloids, amines, betalains), vitamins, and terpenoids (including carotenoids) [70],[71].

### Mechanism of action of antioxidants

The most efficient enzymatic antioxidants are superoxide dismutase (SOD) and catalase (CAT). SOD activity protects cells from free radicals induced injury by catalyzing the dismutation of O_2•_^−^ to O_2_ and to the less-reactive species hydrogen peroxide (H_2_O_2_) [72]. The enzyme CAT on the other hand catalyzes the conversion of H_2_O_2_ to water and molecular oxygen. A cervical cancer based study to examine the relationship between OS and enzymatic antioxidant status in the erythrocytes of adult cervical cancer patients and healthy subjects showed a significant increase in lipid peroxidation and impaired enzymatic antioxidant activities in the erythrocytes of cervical cancer patients [73]. Non-enzymatic antioxidants such as thiol antioxidants and vitamin E also play vital antioxidant roles. Vitamin E prevents lipid peroxidation and aging of cells [69]. Non-protein thiols have a variety of functions in bioreduction and detoxification processes. Intracellular redox homeostasis is regulated by thiol-containing molecules, such as glutathione and thioredoxin [74]. Catalina et al. evaluated the apoptotic effects of Cellfood™ (CF), a nutritional supplement containing deuterium sulphate, minerals, amino acids, and enzymes, on three leukemic cell lines including Jurkat, U937, and K562 cells, and reported that this natural anti-oxidant extracted from the red algae *Lithothamnion calcareum*, modulates cell signalling and apoptosis in cancer cells by activating caspase 3, inducing nucleosomal DNA frangmentation, and altering cell metabolism through down-regulation of HIF-1α and GLUT-1 expression [75]. In a study of the mitochondrial pathway of apoptosis in HepG2 cells, Chang et al. [76] reported that Norcantharidin, the demethylated analog of cantharidin derived from a traditional Chinese medicine, Mylabris, exerts its anti-cancer effects through induction of oxidative stress leading to loss of mitochondrial membrane potential, release of cytochrome c from the mitochondria to the cytosol, down-regulation of Bcl-2, up-regulation of Bax , caspase 3 and caspase 9, and subsequent cleavage of PARP.

### Leukemia

Leukemia is one of the top 10 cancers affecting all races in the United States [77]. Leukemia remains a public health problem though there is a decline in annual death rate when compared with the death rate in 1991 [78]. According to the American Cancer Society estimates, about 1,660,290 new cancer cases and 580,350 cancer deaths occurred in the United States in 2013 and Leukemia account for 48,610 new cases and about 23,720 deaths [78]. Leukemia is cancer of the blood or bone marrow characterized by an abnormal proliferation and circulation of immature clonal hematopoietic cells. Diseases associated with leukemia are commonly referred to as hematological neoplasms and they constitute one of the 10 killer cancers in the US and the most commonly diagnosed cancers and leading causes of cancer death in children aged 0–19 years [79],[80]. In 2011 according to the National Cancer Institute’s Surveillance, Epidemiology, and End Results (SEER) program report, about 302,800 people were living with leukemia in the United States. About 52,380 new cases are estimated in 2014, which accounts for 3.1% of all new cancer cases. Also, 24,090 deaths are expected to occur in the US in 2014 due to leukemia which represents 4.1% of all cancer deaths [81]. Global deaths due to leukemia in 2010 were about 281,500 [82]. In 2000, leukemia accounted for about 3% of the seven million deaths due to cancer and about 0.35% of all deaths from any cause around the world [83]. There is a racial divide in the prevalence of leukemia; white American children are almost twice more likely to develop leukemia than black American children and boys are more likely to be affected than girls. Hispanics under 20 years of age are at the highest risk for leukemia, while whites, Native Americans, Asians, and Alaska Natives are at higher risk than blacks. Around 30 percent more men than women are diagnosed with leukemia and die from the disease [78]. But more than 90% of all leukemias are diagnosed in adults with the peak incidence between 40 and 60 years [84].

### Leukemogenesis

Leukemia develops when hematopoietic stem cells lose the capacity to differentiate normally to mature blood cells at different stages of their maturation and differentiation [85]. Under normal process, all blood cells are originate from blood stem cells with the myeloid pathway producing red blood cells, platelets, and white blood cells, and the lymphoid pathway generating different types of lymphocytes [80]. This hematopoietic system produces the required amount of blood cells during an individual’s lifetime and it involves a carefully balanced mechanism of differentiation, proliferation and self-renewal [86]. The perturbation of this balance due to different cellular conditions and external pressures may lead to irregular differentiation of cell and premature generation of immature/abnormal cells and presence of heterogeneous populations of genetically distinct sub-clones in circulation causing the adverse effects associated with different forms of tumors [87],[88].

### Types of leukemia

**Figure 4:**
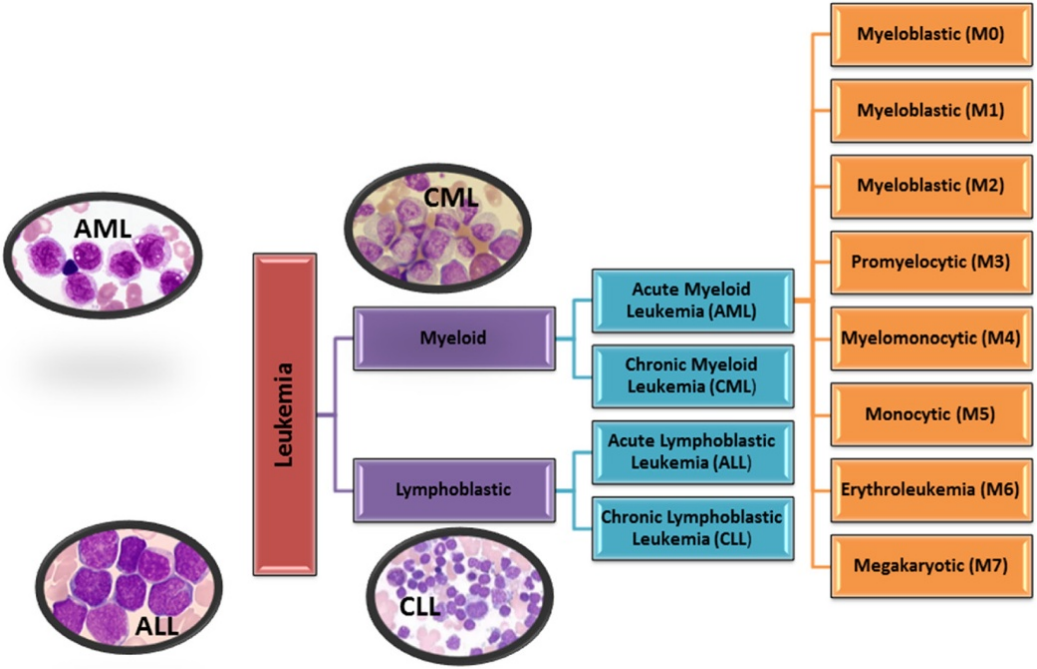
**Major types of leukemia: AML- acute myeloid leukemia; ALL- acute lymphoblastic leukemia; CML- chronic myeloid leukemia; CLL- chronic lymphoblastic leukemia.**

Leukemia are classified based on; onset (acute or chronic), the affected blood cell type (lymphoblastic/lymphocytic or myeloid/myelogenous), the maturity stage of the blood cell and phenotypic expression of the disease. There are four major clinical types of leukemia and there also different pathological subtypes as shown in Table 1. The common types of leukemia are; acute myeloid leukemia (AML), acute lymphoblastic leukemia (ALL), chronic myeloid leukemia (CML), chronic lymphocytic leukemia (CLL), and acute promyelocytic leukemia (APL) [87] as illustrated in Figure 4. AML is further divided into eight subtypes based on the cell the leukemia developed from: Myeloblastic (M0), Myeloblastic (M1) - without maturation, Myeloblastic (M2) - with maturation, Promyeloctic (M3), Myelomonocytic (M4), Monocytic (M5), Erythroleukemia (M6) and Megakaryocytic (M7) [89]. Other sub-types include; hairy cell leukemia, chronic myelomonocytic leukemia (CMML), juvenile myelomonocytic leukemia (JMML) is seen mostly in children and it could be cured using hematopoietic stem cell transplantation (HSCT) [79],[90],[91].

**Table 1:** **Differentiating characteristics of the four types of leukemia**

Type/Age at onset (Yr.)	Gender predilection	Racial predilection	Cell of origin	Specific markers
**Acute Lymphocytic (ALL) <15**	Males	Caucasian	B-cell	CALLA+
				Hyperdiploidy
				TDT+
**Acute Myelogenous (AML) 15 - 39**	Equal incidence	None	Myeloblast	TDT-
			Myelocyte	t(9;22)
			Promyelocyte	t(15;17)
			Myelomonocyte	
**Chronic Myelogenous (CML) > 50**	Males	None	Myeloid cell	Ph^1^ chromosome

### Acute myeloid leukemia

Acute myeloid leukemia (AML) known by different names such as acute myelogenous leukemia, acute granulocyctic leukemia or acute non-lymphocytic leukemia is a malignant neoplasm that originates in the cells within the bone marrow. AML can be divided into five main types: AML with genetic abnormalities (e.g. t[8;21], t(15;17), t(9;22) ), AML with FLT3 mutation, AML with multilineage dysplasia, therapy-related AML, and uncategorized AML [84]. However, World Health Organization (WHO) compresses AML types into three subgroups: (1) AML with recurrent genetic abnormalities, (2) AML with multilineage dysplasia, and (3) AML and MDS (myelodysplastic syndromes), therapy related [92]. AML is the second most common type of leukemia in children [93] and it affects heterogeneous group of tumor cell populations including myeloblast, myelocyte, promyelocyte, and myelomonocyte. It is described as acute because AML progresses quickly without treatment. Primitive myeloid cells are not well developed and cannot carry out the normal functions of the blood cell [79]. As a result cellular and molecular activities malfunction leading to DNA damage, increased proliferation, deficient cell death, genetic instability and ROS-induced OS [10],[94],[95]. MDS are malignant tumors that closely resemble AML and may develop to AML [96]. MDS are characterized by cytopenias which predisposes to infection, bleeding, and death [96]. Most AML can be characterized by the expression of the common acute lymphoblastic leukemia antigen (CALLA) which is a 749-amino acid type II integral membrane protein [97]. WHO recommends using the following cytogenetics parameters to define AML to avoid ambiguity, mischaracterization and misdiagnosis of AML; patients with the specific recurring cytogenetic abnormalities t(8;21)(q22;q22), inv(16)(p13q22) or t(16;16)(p13;q22), and t(15;17)(q22;q12) regardless of the blast percentage [84]. Both genetic predisposition and environmental risk factors such as radiation, cigarette smoking and exposure to other environmental carcinogens have been linked to AML pathogenesis. A previous study by Zhuo et al. [98] pointed out that CYP1A1 MspI polymorphism might be a possible risk factor for AML in Asian populations. Prognosis of AML is generally poor but best prognosis is with bone marrow transplant [99]. Acute leukemia may also have blast cells that are Sudan Black and terminal deoxynucleotidyl transferase (TdT) positive [100]. The survival rate of AML is very slim necessitating development of more effective treatment therapies [101],[102]. Treatment for AML may include chemotherapy, radiation therapy, stem cell transplant and/or immunotherapy [81],[89].

### Chronic Myelogenous Leukemia (CML)

CML also referred to as chronic myeloid, chronic myelocytic or chronic granulocytic leukemia, is a clonal myeloproliferative disorder in which transformed, primitive hematopoietic progenitor cells are pushed into circulation. CML is characterized by increased proliferation of granulocytic cells without loss in their capacity to differentiate. CML is characterized by the Philadelphia (Ph^1^) chromosome and it is the first human disease malignancy associated with a gene translocation in which a specific abnormality of the karyotype could be linked to pathogenic processes of leukemogenesis [103]. The Philadelphia (Ph^1^) chromosome is created from the bcr-abl fusion gene, which is a result of the reciprocal translocation between the *abl* oncogene *on* the long arm of chromosome 9 and the *bcr* region on the long arm of chromosome 22, t(9;22)(q34;q11). The bcr-abl fusion gene is seen in more than 90% of CML cases [104],[105]. Prognosis is generally poor and it is worse if there is no Ph^1^ chromosome. In CML the chronic phase is often followed by an accelerated blastic phase, a more acute disease phase, which is generally fatal [103]. In a study of CML pathogenesis, Long et al. [106] evaluated the role of the Hedgehog (Hh) signaling pathway, and reported that Hh-related genes such as Sonic hedgehog (Shh), Smoothened (Smo), and Gli1 genes were significantly upregulated in CML patients when compared with normal people. They concluded that Hh signalling maybe associated with CML progression [106]. Treatment for CML may include radiation therapy, chemotherapy, stem cell transplant and/or immunotherapy. A common treatment for chronic leukemias is oral chemotherapy such as Gleevec (imatinib), Sprycel (dasatinib) and Tasigna (nilotinib) [89].

### Chronic Myelomonocytic Leukemia (CMML)

CMML is an aggressive malignancy characterized by ineffective hematopoiesis and peripheral monocytosis. It was previously classified as a subtype of the myelodysplastic syndromes (MDSs) but was recently demonstrated to be a distinct entity with distinct characteristics [107]. However, it is placed under mixed myelodysplastic/myeloproliferative disease in the WHO classification [108]. About 20 to 40 percent of CMML patients have chromosomal abnormalities with 1 to 4 percent having translocation involving the PDGFR-β and TEL genes [90]. Chemotherapy with imatinib has been successful in the treatment or patients with PDGFR-β and TEL gene mutation [109].

### Acute Promyelocytic Leukemia (APL)

APL is a form of acute myeloid leukemia in which abnormal promyelocytes predominate and it can affect both adults and children but mostly children [110]. The over production of promyelocytes leads to a shortage of normal white blood cells, red blood cells and platelets in circulation, which causes many of the signs and symptoms observed in APL. General signs and symptoms may occur as fever, loss of appetite, and weight loss but disseminated intravascular coagulation is a common symptom and could be life-threatening. Other signs of the malignancy include leukopenia, susceptibility to developing bruises, small red dots under the skin (petechiae), nosebleeds, bleeding from the gums, blood in the urine (hematuria), or excessive menstrual bleeding [111], low number of red blood cells (anemia), and excessive tiredness (fatigue). Some patients experience bones and joints pains when the leukemic cells spread to the bones and joints [110]. Genetic studies show that cells from most patients have a balanced reciprocal translocation between chromosomes 15 and 17 [112], which generates a fusion transcript joining the *PML*(promyelocyte) and *RAR*-α (retinoic acid receptor-α) genes [113]. The promyelocytic leukemia gene (PML) involved in the t(15;17) (q22;q12) translocation is a growth suppressor and pro-apoptotic factor [114],[115]. Disruption of the PML gene by the t(15:17) translocation in APL could be critical in leukemogenesis because accelerated cell proliferation was observed when the PML gene was knocked-out [116],[117]. APL is most often diagnosed around age 40, although it can be diagnosed at any age. Prognosis is poorer in adults than in children but prognosis is better than in AML and it is curable in children [118]. A combination of All-*trans* retinoic acid (ATRA) and arsenic trioxide (ATO) has been effective in treating APL especially in newly diagnosed patients. However, ATRA with anthracycline-based chemotherapy for induction and consolidation and additional use of low dose maintenance ATRA is considered as the standard treatment protocol [110]. ATRA has been reported to exert its therapeutic action against APL cancer through induction of cell differentiation via mechanisms that include degradation of PML-RARA gene [119] and inhibition of arachidonic acid metabolic pathway in other cancer cells [119].

### Acute Lymphoblastic Leukemia (ALL)

ALL is a disease characterized by uncontrolled proliferation and maturation arrest of lymphoid progenitor cells in bone marrow resulting in an excess of malignant cells. The lymphoblasts replace the normal marrow elements, resulting in a marked decrease in the production of normal blood cells leading to varying degrees of anemia, thrombocytopenia, and neutropenia [120]. ALL is the most common cancer found in children and it accounts for more than 50% of childhood hematopoietic malignancies. But it is relatively rare in adults, accounting for only 2–3% of hematopoietic malignancies [120]. Abnormal expression of genes, which is usually a result of chromosomal translocations, is suggested as one of the causes of ALL. The examination of the cytogenetic lesion in Ph(+) in ALL shows that the translocation of most cases of ALL with break point in the minor cluster region (m-BCR) give rise to (P190) fusion protein [121]. A previous in-vitro study using inhibitors of glycogen synthase kinase-3β (GSK-3β) found that it significantly accumulates in the nuclei of ALL cells compared to control cells; leading to a downregulation of NF-κB-target Survivin gene and promotion of apoptosis in ALL cells in vitro [122]. It is a curable disease with an expected long term survival rate of at least 70%, when treated with modern therapeutic regimens. A common chemotherapy treatment for ALL begins with induction chemotherapy, in which a combination of drugs is used to destroy as many leukemia cells as possible and bring blood counts to normal [123]. This is followed by consolidation chemotherapy, to destroy any remaining leukemia cells that cannot be seen in the blood or bone marrow. Patients with ALL may also receive maintenance chemotherapy. This less intensive course of chemotherapy is used to reduce the risk of the disease recurring after treatment has finished [123].

### Chronic Lymphocytic Leukemia (CLL)

CLL starts in B-cells in the bone marrow before invading the blood. Leukemia cells take a long time to accumulate in the peripheral blood, bone marrow, and lymphoid tissues and may take a few years before symptoms start showing up in many people [124]. In the United States about 15,720 new cases of CLL and 4,600 deaths will occur in 2014 [125]. Most of CLL patients are the elderly, with a median age at diagnosis of 72 years with more males than females being affected. Also, more Caucasians than African-Americans have CLL and rarely seen among Hispanics, Native Americans, and Asian populations [126]. The prognosis is poor and it is the only leukemia with a possible genetic predisposition [124],[126]. Features of CLL include hypogammaglobulinemia and recurrent infection due to compromised humoral and cellular immunity [127]. Another feature observed in CLL is Trisomy 12 and it is one of chromosomal abnormalities frequently seen in B-cell CLL [128]. OS has been recognized to play a role in CLL development and treatment response. A recent study evaluating ROS-related damages in lymphocytes from patients with monoclonal B-lymphocytosis and CLL reported increased levels of oxidatively modified DNA and lipids in the sera of untreated CLL patients due to increased oxidative phosphorylation in CLL cells. Furthermore, CLL cells adapted to intrinsic OS by up-regulating the stress-responsive heme-oxygenase-1 (HO-1) [117]. Ibrutinib is approved for the treatment of patients with CLL who have received at least one prior therapy. It is also approved for relapsed/refractory mantle-cell lymphoma patients [129]. Hairy cell leukemia is a type of chronic lymphocytic leukemia [130].

### Leukemia and reactive oxygen species

It is well recognized that oxidants play a role in several stages of carcinogenesis [53]. OS is associated with numerous pathological phenomena, including infection, inflammation, ultraviolet- and γ-irradiation, increased mutation frequency [79] and acute promyelocytic leukemia [131]. Increased free radical generation, especially superoxide anion in leukemia patients and increased antioxidant defense enzymes, which is an adaptive protective response, are indicative of mild OS [43]. Several studies have implicated OS as a factor in carcinogenesis [132],[133]. Some endogenous oxidants are considered as important naturally occurring carcinogens and may contribute to several stages of malignant transformation [134],[135]. ROS can induce genetic mutations as well as chromosomal alterations and thus contribute to cancer development in multistep carcinogenesis [133],[136]. ROS also can trigger intercellular secondary messengers and thus modulate various aspects of cellular functions including proliferation, apoptosis, and gene expression [137]. ROS initiate carcinogenesis by activating kinases, denaturing DNA through induction of poly ADP-ribosylation of chromosomal proteins [134]. Cell damage from oxygen free radicals (OFR) is ubiquitous and may be significant in the expansion of tumor clones and the acquisition of malignant properties [135]. Stress-activated signaling cascades are affected by altered redox potential due to ROS formed by exogenous genotoxic agents such as irradiation, inflammatory cytokines and chemical carcinogens. ROS and altered redox potential can be considered as the primary intracellular changes which regulate protein kinases, thus serving as an important cellular component linking external stimuli with signal transduction in stress response [137]. As observed earlier, various antioxidants are decreased in cancer, SOD and CAT activities were decreased in ALL patients. Reduced CAT activity was observed in just diagnosed patients and patients in both treatment groups suggesting a disturbance of the protective role of these enzymes against free radicals in ALL and chronic lymphocytic leukemia (CLL) [10],[138]. Nishiura et al. reported elevated serum SOD activity in acute leukemia and indicated that regression of the leukemia was accompanied by a decrease in the serum level of SOD [139]. Likewise, there is GSH depletion in lymphocytes isolated from the blood of patients with CLL [140]. These findings suggest that there are alterations in the enzymatic antioxidant defenses, which can interfere in the direct removal of free radicals (pro-oxidants) and in the protection for biological sites [141].

### Oxidative stress in leukemia treatment

**Figure 5:**
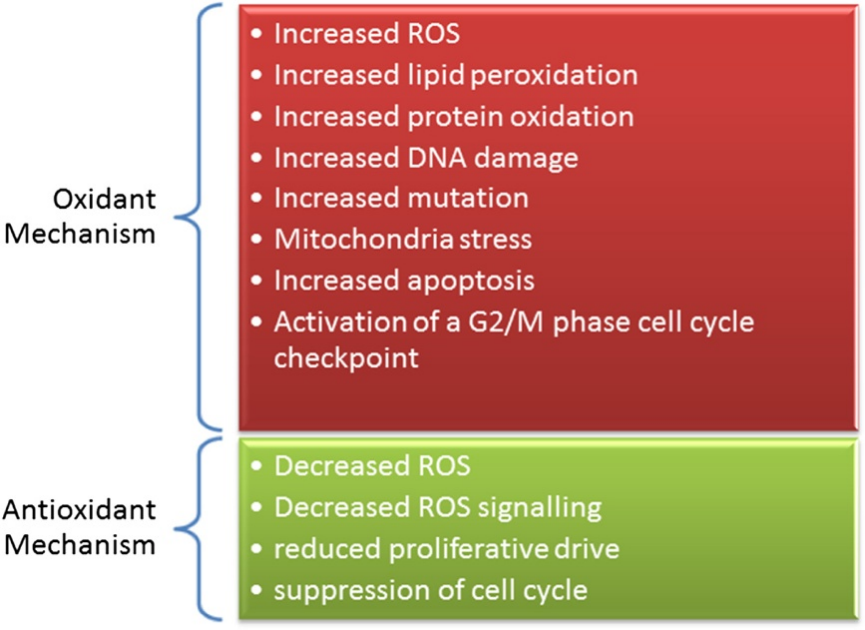
**Antioxidant treatment versus pro-oxidant treatment as a therapy for hematologic malignancy.**

Traditionally, the appropriate treatment for any leukemia disease depends mainly on the type of leukemia, age, and general health of the patient [89]. The current cytotoxic drugs used in standard leukemia therapy are designed to attack DNA replication process within malignant cells, and it does not discriminate between malignant and non-malignant cells since it targets cell proliferation [142]. Adding to the toxic side effects of standard chemotherapy is the issues of drug resistance from quiescent clones [143]. These make discovery of new treatments measures for leukemia very imperative. Targeting ROS level could be a novel approach since ROS levels are higher in malignant cell than in non-malignant cells. Despite the negative effect of oxidants some of their mechanisms of action have found application in the treatment of malignant diseases [6]. Conversely, there are reports showing that antioxidative conditions promote some carcinogenic processes [144]. Both antioxidant and oxidants processes are being explored for treatment of hematologic malignancy. Lau and co-authors in their review discussed the potential applications of ROS in leukemia treatment [6]. As illustrated in Figure 5, there two approaches applied in using ROS for leukemia treatment; oxidant treatment and anti-oxidant treatment. The oxidant approaches may cause cell death by: increasing ROS, lipid peroxidation, protein oxidation and mutation, increasing mitochondria stress, apoptosis, and activation of a G2/M phase cell cycle checkpoint [145]. The antioxidant mechanism uses the following reactions to balance the negative effect of the oxidant processes: reduced ROS signalling, reduced proliferative drive, and suppression of cell cycle which may reduce tumor burden and buffer nonmalignant cells from oxidative damage [146]. A combination of antioxidants and chemotherapeutic agents have promising synergistic effect, however, some argue that suppression of cell cycle and antagonism of chemotherapy-induced ROS may also affect treatment efficacy [147]. Vitamin antioxidants have been shown to reduce the risk of various cancers by suppressing the state of OS [148],[149]. Likewise, antioxidant vitamins C and E from supplements have shown promises in reducing risk of ovarian cancer [149].

Pro-oxidant treatment may cause depletion of antioxidant defenses leading to more production of ROS beyond the level produced by the malignant cell [31]. Although it may be difficult it is very important to determine the optimum level of ROS that will be most efficient and effective in the treatment of Leukemia, only a thin line separates the beneficial level and deleterious level of ROS [150]. For instance when the intrinsic stress that is already present in malignant cells is doubled by the treatment-induced OS malignant cells can be sensitized to mainstay treatments or initiate the apoptotic pathway [31]. It can conversely trigger lipid peroxidation, oxidation of redox-sensitive residues within proteins, and DNA oxidation leading to increased tumor burden [147],[150]. As observed with proxidants, extramitochondrial antioxidants such as vitamin C (ascorbic acid) can act both as prooxidant and as antioxidant [144].

Bortezomib-induced oxidative cell injury function at a proximal point in the cell death cascade to attack and disrupt cytoprotective ERK1/2 signaling. The JNK pathway, is activated followed by induction of mitochondrial dysfunction, caspase activation, and apoptotic cell death [151]. Anthracycline daunorubicin is widely used in the treatment of acute nonlymphocytic leukemia and oxidative stress is one of the triggers of the body’s response to this drug [152]. In a study from our laboratory we demonstrated that dietary supplement such as garlic induces cytotoxicity and apoptosis in HL-60 cells through phosphatidylserine externalization, caspase-3 activation, and nucleosomal DNA fragmentation associated with the formation of malondialdehyde, a by-product of lipid peroxidation and biomarker of OS [153].

Application of antioxidant principles may illicit same effect, for example inhibition of intracellular antioxidants such as GSH [154] and heme oxygenase-1 (HO-1) [155]. Isothiocyanates and adaphostine are other pro-oxidant drugs. Isothiocyanates act by depleting GSH pools, and efficiently kill fludarabine-resistant chronic lymphocytic leukemia (CLL) cells [156] and imatinib-resistant CML cells, [157] selectively without attacking normal hematopoietic cells. Adaphostine, a tyrphostin kinase inhibitor is able to induce up-regulation of ROS and cause DNA damage-induced apoptosis in BCR-ABL–expressing CML [158]. Other leukemic drugs that have pro-oxidant properties include arsenic trioxide (ATO) which is currently used for treatment of relapsed APL which may function by inhibiting thioredoxin [159], inducing ROS production [160] or NOX activation [161]. However, Jeanne and co-researchers suggest that ATO-induced ROS production plays a critical role in degradation of PML-RARα fusion proteins in ATO-treated APL cells [162]. Isothiocyanates when used together with ATO were effective in killing CML- and AML-derived cell lines in vitro [163]. In a recent in vitro study, we demonstrated that OS plays a key role in ATO-induced mitochondrial pathway of apoptosis in HL-60 cells. We discovered that this apoptotic signaling is associated with DNA damage, change in mitochondrial membrane potential, activation and translocation of pro-apoptotic proteins, and down regulation of anti-apoptotic proteins [2]. These findings are in support of previous research from our laboratory reporting phosphatidylserine externalization and caspase 3 activation and nucleosomal DNA fragmentation in HL-60 cells exposed to ATO [164]. A recent study from our laboratory has also demonstrated that vitamin D3 (Vit D3) potentiates the antitumor effect of ATO in HL-60 cells. This potentiation is mediated at least in part, through induction of phosphatidylserine externalization and nucleosomal DNA fragmentation [165]. Also, we previously reported similar potentiation of ATO effect by vitamin C [166],[167]. These findings highlight the potential impact of Vit C and Vit D3 in promoting the pharmacological effect of ATO, suggesting a possible future role of Vit C/Vit D3/ATO combination therapy in patients with acute promyelocytic leukemia.

Antioxidant molecules can be used to suppress the high levels of ROS observed in some cancer cells. For example targeting NOX4-derived ROS which promotes survival in some pancreatic cancers could be effective in growth arrest [168]. Also, antioxidant treatment may alleviate chemotherapy-related toxicity, reducing the requirement for dose reduction in some patients and allowing an increased proportion of patients to complete their therapy [169]. Another study demonstrated a trend of longer clinical progression-free survival and overall survival in CML patients when they were treated with vitamin A in combination with standard chemotherapy, although this trend was not significant [170]. Figure 5 illustrates the involvement of oxidant and antioxidant treatment in the control of leukemia cancer.

## Conclusions

OS has both beneficial and negative effect on leukemogenesis. Most of the genes that regulate the redox processes have double edged sword activities. This makes it difficult to determine the optimum point to harness the therapeutic attributes because only a very thin line separates the oncogenic properties from the tumor suppressive activity. A better understanding of the relationship between OS and leukemogenesis will give more insight on how to ameliorate its deleterious effect and how to tap unto the beneficial attributes. It is known that ROS causes nonspecific oxidative damage to biomolecules in myeloid cells, under this condition there is a persistent increase in ROS level and depletion of the cell’s antioxidant defenses culminating in cancer induction. Another process is the hyperactivation of ROS signaling pathway. Significant progress has been made in developing therapeutic measures for various types of leukemia and some can be cured while treatment for some such as, myeloid leukemia are still difficult. New effective therapeutic strategies are needed. Increased OS in some myeloid leukemia may be a promising therapeutic target. Despite the negative effect of OS to the cell, tapping into the possible application of its activities for therapeutic capabilities is worth pursuing because even the current leukemia treatment drugs have cytotoxic effects as well. Further studies are required to determine the source and species of ROS generated by leukemic cells and whether the ROS that has therapeutic effects originate from the normal cell metabolism or from the malignant cell population. The knowledge that OS induces lipid peroxidation and protein carbonylation by inactivating antioxidant enzymes could be used to determine an efficient and effective way to boost endogenous production and incorporation of antioxidants into diets to neutralize the free radical produced by cellular metabolisms. This may serve as potent prophylaxis for various leukemia since most cancers develop under OS environment. It is heartening that some drugs based on oxidative mechanisms are on clinical trial stage. Further studies are needed to understand the effectiveness, long time effect and consequences of using such drugs.

## Authors’ original submitted files for images

Below are the links to the authors’ original submitted files for images. Authors’ original file for figure 1 Authors’ original file for figure 2 Authors’ original file for figure 3 Authors’ original file for figure 4 Authors’ original file for figure 5

## Abbreviations

ALA: Alpha lipoic acid
ALL: Acute lymphoblastic leukemia
AML: Acute myeloid leukemia
Ang II: Angiotensin II
ATO: Arsenic trioxide
ATRA: All-*trans* retinoic acid
CALLA: Common acute lymphoblastic leukemia antigen
CAT: Catalase
CLL: Chronic lymphoblastic leukemia
CML: Chronic myeloid leukemia
CMML: Chronic myelomonocytic leukemia
CoQ10: Coenzyme Q10
GPx: Glutathione peroxidase
GSH: Glutathion
GSK-3β: Glycogen synthase kinase-3β
Hh: Hedgehog
HO-1: Heme-oxygenase-1
HSC: Hematopoietic stem cell
HSCT: Hematopoietic stem cell transplantation
JMML: Juvenile myelomonocytic leukemia
MBL: Monoclonal B-cell lymphocytosis
MDS: Myelodysplastic syndromes
MMP-1: Matrix metallopeptidase 1
NADPH: Nicotinamide adenine dinucleotide phosphate
NCI: National Cancer Institute
OFR: Oxygen free radicals
OS: Oxidative stress
PML: Promyelocytic leukemia gene
PPARγ: Peroxisome proliferator-activated receptor gamma
RNI: Reactive nitrogen intermediate
RNS: Reactive nitrogen species
ROS: Reactive oxygen species
SEER: Ssurveillance, epidemiology, and end results
Shh: Sonic hedgehog
SOD: Superoxide dismutase
STAT3: Signal transducer and activator of transcription 3
SYK: Spleen tyrosine kinase
TBARS: Thiobarbituric acid reactive substances
TdT: Deoxynucleotidyl transferase
WHO: World Health Organization
XO: Xanthine oxidase
