# Supplementary figures and images for: Dual effect of oxidative stress on leukemia cancer induction and treatment
Source: J Exp Clin Cancer Res. 2014 Dec 18;33:106. doi: 10.1186/s13046-014-0106-5 (PMC4320640; doi:10.1186/s13046-014-0106-5)

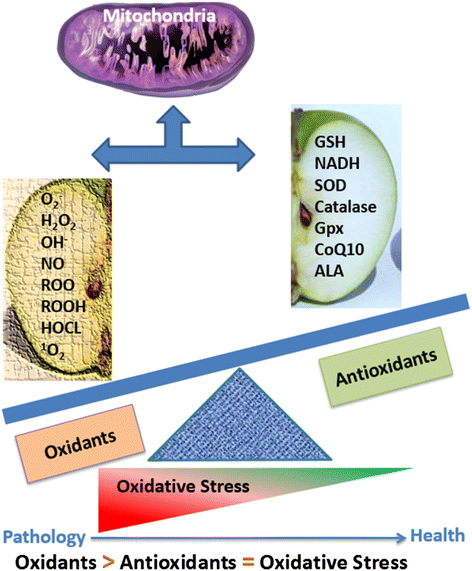

Supplement: Supplementary file 1 — Authors’ original file for figure 1 [file 13046_2014_106_MOESM1_ESM.gif]

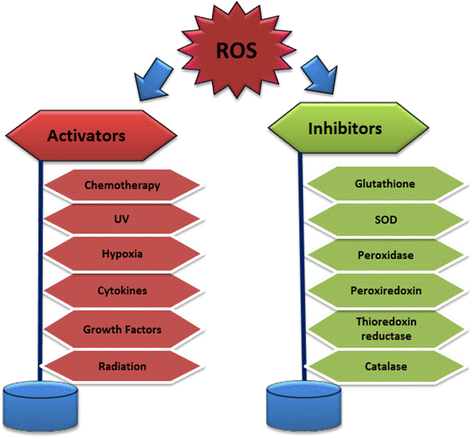

Supplement: Supplementary file 2 — Authors’ original file for figure 2 [file 13046_2014_106_MOESM2_ESM.gif]

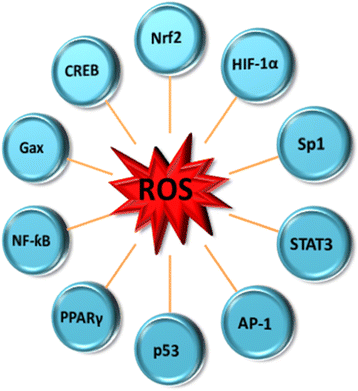

Supplement: Supplementary file 3 — Authors’ original file for figure 3 [file 13046_2014_106_MOESM3_ESM.gif]

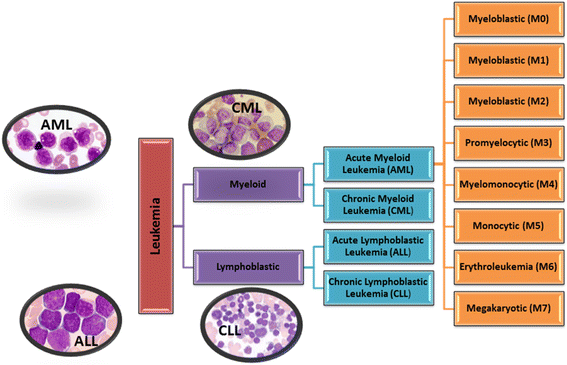

Supplement: Supplementary file 4 — Authors’ original file for figure 4 [file 13046_2014_106_MOESM4_ESM.gif]

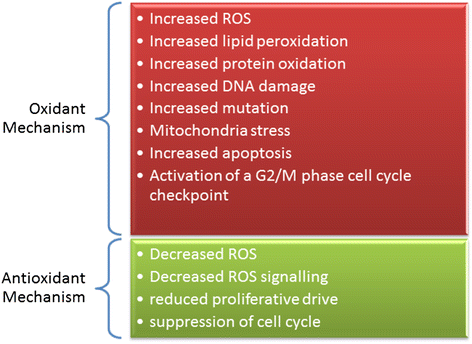

Supplement: Supplementary file 5 — Authors’ original file for figure 5 [file 13046_2014_106_MOESM5_ESM.gif]
